# Age-dependent changes in autophosphorylation of alpha calcium/calmodulin dependent kinase II in hippocampus and amygdala after contextual fear conditioning

**DOI:** 10.1016/j.brainresbull.2017.06.012

**Published:** 2017-09

**Authors:** Ton Fang, Kamillia Kasbi, Stephanie Rothe, Wajeeha Aziz, K. Peter Giese

**Affiliations:** a Maurice Wohl Clinical Neuroscience Institute, Department of Basic and Clinical Neuroscience, King’s College London, United Kingdom; b University of Sussex, Sussex House, Falmer Brighton, BN1 9RH, United Kingdom

**Keywords:** Long-term potentiation, CaMKII, Amygdala, Hippocampus, Contextual fear conditioning, Immunohistochemistry

## Abstract

The hippocampus and amygdala are essential brain regions responsible for contextual fear conditioning (CFC). The autophosphorylation of alpha calcium-calmodulin kinase II (αCaMKII) at threonine-286 (T286) is a critical step implicated in long-term potentiation (LTP), learning and memory. However, the changes in αCaMKII levels with aging and training in associated brain regions are not fully understood. Here, we studied how aging and training affect the levels of phosphorylated (T286) and proportion of phosphorylated:total αCaMKII in the hippocampus and amygdala. Young and aged mice, naïve (untrained) and trained in CFC, were analysed by immunohistochemistry for the levels of total and phosphorylated αCaMKII in the hippocampus and amygdala. We found that two hours after CFC training, young mice exhibited a higher level of phosphorylated and increased ratio of phosphorylated:total αCaMKII in hippocampal CA3 stratum radiatum. Furthermore, aged untrained mice showed a higher ratio of phosphorylated:total αCaMKII in the CA3 region of the hippocampus when compared to the young untrained group. No effect of training or aging were seen in the central, lateral and basolateral amygdala regions, for both phosphorylated and ratio of phosphorylated:total αCaMKII. These results show that aging impairs the training-induced upregulation of autophosphorylated (T286) αCaMKII in the CA3 stratum radiatum of the hippocampus. This indicates that distinct age-related mechanisms underlie CFC that may rely more heavily on NMDA receptor-dependent plasticity in young age.

## Highlights

• Conditioning-induced increase of phospho-αCaMKII in area CA3 is age-dependent.
• Aging increases the ratio of phosphor/total αCaMKII in area CA3.
• Conditioning-induced decrease of αCaMKII in lateral amygdala is age-dependent.
• Aging does not impact on αCaMKII in central and basolateral amygdala.

## 1 Introduction

In the hippocampus, long-term potentiation (LTP) has been implicated as one of the memory mechanisms (Giese, 2012). Autophosphorylation at the threonine-286 (T286) of alpha calcium/calmodulin-dependent kinase II (αCaMKII) is critical to inducing NMDA receptor-dependent LTP in hippocampal area CA1 (Lisman et al., 2012, Giese and Mizuno, 2013). The absence of this LTP combined with the impairment of spatial and contextual fear memories in knock-in mutant mice lacking the T286 autophosphorylation suggest that NMDA receptor-dependent LTP is fundamentally important for hippocampal learning and memory (Giese and Mizuno, 2013). NMDA receptor-dependent LTP results from the depolarisation of synapses and removal of the magnesium block from NMDA receptors, allowing for calcium ions to infiltrate the post-synaptic receptor, resulting in αCaMKII autophosphorylation and neuroplasticity changes (Lisman et al., 2012). Unlike NMDA receptor-dependent LTP, NMDA receptor-independent LTP occurs more predominantly in aged animals in the hippocampus and involves the activation of L-type voltage-gated calcium channels (VGCC) (Boric et al., 2008), possibly because expression of VGCC increases with age (Oh et al., 2016). Whilst VGCCs can activate αCaMKII autophosphorylation (Pasek et al., 2015), it is unlikely that VGCC-dependent LTP is induced during hippocampus-dependent memory formation in young age (Whitlock et al., 2006, Gruart et al., 2015). However, it is unknown whether in old age VGCC-dependent LTP contributes to memory formation.

Contextual fear conditioning is a hippocampus-dependent memory task that involves the amygdala to form and retain memory, after invoking the mice’s fear response in the form of freezing through training with electrical foot shocks (Maren et al., 2013). The tri-synaptic pathway in the hippocampus is critical to memory where input from the entorhinal cortex is processed and filtered to the dentate gyrus, CA3 and then the CA1 brain regions. This predominantly excitatory pathway is modulated by inputs from the amygdala to mediate learning through changes in neuronal plasticity (Shechner et al., 2014). The amygdala, which is part of the limbic system plays a role in this emotional learning and is the brain region where connections from sensory cortical inputs are converted into specific autonomic and behavioural responses, such as the fear response demonstrated during contextual fear conditioning (Davis, 1994, Schafe et al., 2005).

Aging impairs contextual fear and spatial memory (Murphy et al., 2004, Peters et al., 2014). A possible explanation for this deterioration may come from age-related difficulties in activating NMDA receptors, raising the threshold for NMDA receptor-dependent LTP induction (Murphy et al., 2004, Bonhaus et al., 1990, Kumar, 2015, Deupree et al., 1993, Moore et al., 1993, Diana et al., 1995, Barnes et al., 1996, Kumar et al., 2007). Furthermore, aging encourages NMDA receptor-independent methods for LTP induction using VGCC channels in the hippocampus (Boric et al., 2008). Moreover, recent studies have shown after complete lesioning of the hippocampus, contextual fear memories can still be formed after multiple training trials (Wiltgen et al., 2006), suggesting that other brain regions such as the amygdala may be involved in learning and memory. Therefore, it is important to study how aging can affect critical molecular mechanisms such as autophosphorylation of αCaMKII after CFC in associated brain regions. We systematically analysed the phosphorylated T286 (active) and total levels of αCaMKII in the hippocampus and amygdala after contextual fear training in young and aged mice.

## 2 Materials and methods

### 2.1 Subjects

Experiments were conducted using female C57BL/6J inbred mice (Harlan, NL), aged mice (n = 10) were 18 months and young mice (n = 10) 3 months old. All work-involving mice were conducted in accordance with the UK Animals Scientific Procedures Act 1986.

### 2.2 Training

Half of the mice from each age group was randomly allocated to contextual fear conditioning (CFC), creating four unique groups; young trained (YT, n = 5), young untrained (YU, n = 5), aged trained (AT, n = 5) and aged untrained (AU, n = 5). Mice dedicated to training were placed one at a time in an enclosed observational chamber (MedAssociates), with their first shock (0.7 mA) lasting 2 s, being administered 148 s after insertion. Subsequent shocks were applied at 90 s intervals and this was repeated four times (five shocks in total), before the mice were returned to their habitat 30 s after their final shock. This conditioning protocol led to similar levels of 24-h contextual fear memory in young and aged mice (Aziz et al., in preparation).

### 2.3 Tissue preparation

Mice were perfused and brains isolated two hours post-conditioning, brains were post-fixed in 4% paraformaldehyde, 30% sucrose and flash frozen following protocols from previous studies (Radwanska et al., 2011). 40 μm thick slices were achieved using a cryotome (Leica Biosystems, DE) and slices at −1.50 mm bregma point were isolated and stained following a previous method for phosphorylated αCaMKII (T286) (Ouyang et al., 1997) and a modified protocol for total αCaMKII dilutions (Cox and Racca, 2013), antibodies shown in Table 1. Antibody specificity was determined by staining of a sample with no primary antibodies. Staining for phosphorylated and total αCaMKII was conducted on adjacent brain samples. Images were acquired for two slices per animal using Axio Imager 2 with Apotome (Zeiss, DE) of the hippocampus (CA3 stratum radiatum), amygdala (basolateral, lateral and central regions) and hippocampus (CA1 stratum oriens) to use as a background.

**Table 1:** Primary and secondary antibodies used for immunohistochemistry staining for phosphorylated and total αCaMKII. Antibody concentrations, name and conditions for staining displayed.

Experiment type	Antibody type	Name	Species	Special Conditions	Dilution
Phosphorylated αCaMKII (T286)	Primary	ABCAM, UK (AB5683)	Rabbit polyclonal	18 h rt	1:300
	Secondary	Life Tech, US (A11034)	Goat anti-Rabbit	2 h rt	1:2000
Total αCaMKII	Primary	Abnova Corp., ROC (MAB8699)	Mouse monoclonal	72 h 4 °C	1:2000
	Secondary	Life Tech, US (A11004)	Goat anti-mouse	2 h rt	1:500

### 2.4 Data analysis

Mean αCaMKII density was calculated by taking measurements from three identical rectangular areas on images and taken as a ratio of mean background density using the corresponding mean CA1 stratum oriens αCaMKII value. If mean background density was indeterminate for a particular slice due to damage during preparation, then the background value of the corresponding slice for that animal was used. There were no instances where both background values for the same animal was missing. Ratios after background calculations of the two slices for each brain areas were averaged to give mean density per animal, for both phosphorylated (T286) and total αCaMKII. The proportion of αCaMKII that was phosphorylated compared to the total was calculated as a ratio, by dividing phosphorylated with total values for each animal. An average group value was used to treat missing values and allowed for a ratio for each animal to be obtained. All data was standardised accordingly with YU values by dividing with the corresponding YU data. Graphs were plotted as mean ± standard error of mean on GraphPad Prism v7.0, US.

### 2.5 Statistical analysis

Two-way ANOVA was conducted for all animal groups and Tukey post-hoc analysis was undertaken to signify specific differences in total, phosphorylated and ratio of phosphorylated:total αCaMKII, between young and aged mice in different brain regions. For the ratio of phosphorylated:total αCaMKII in CA3 hippocampus, lateral and central amygdala, original data was not normally distributed and instead a two-way ANOVA was conducted further to log transformation. All other outcome variables were found to be normally distributed and of equal variance. Statistical tests were carried out on SigmaPlot v13.0, US.

## 3 Results

**Fig. 1:**
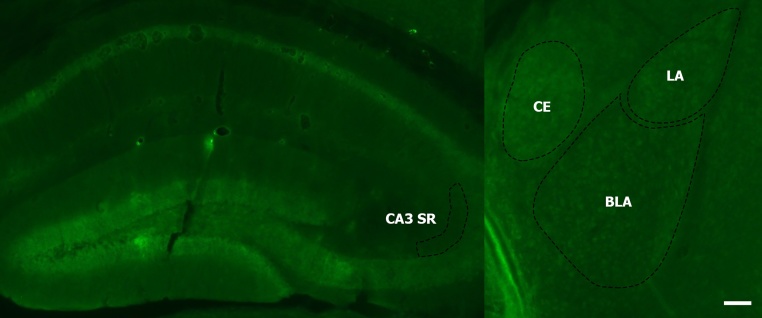
Low magnification images of the brain regions stained with pCaMKII antibody. Hippocampus (left) showing CA3 stratum radiatum (SR) and amygdala (right) showing lateral (LA), basolateral (BLA) and central (CE) regions as indicated. Images stained using eGFP (green). Scale bar, 40 μm. (For interpretation of the references to colour in this figure legend, the reader is referred to the web version of this article.)

The autophosphorylation (T286) of αCaMKII is essential for NMDA receptor-dependent LTP (Lisman et al., 2012, Giese and Mizuno, 2013), and also occurs after the induction of VGCC-dependent LTP (Pasek et al., 2015), lasting for several hours (Irvine et al., 2006). We tested whether aging and contextual fear conditioning can alter the levels of phosphorylated αCaMKII in hippocampus and amygdala nuclei (Fig. 1), by analysing the T286 phosphorylated (activated) and total levels of αCaMKII. It was previously shown that phosphorylation of αCaMKII occurs two hours after contextual fear conditioning (Atkins et al., 1998). Similarly, in our study we have chosen a two-hour time point to study changes in phosphorylated αCaMKII for varying brain regions.

**Fig. 2:**
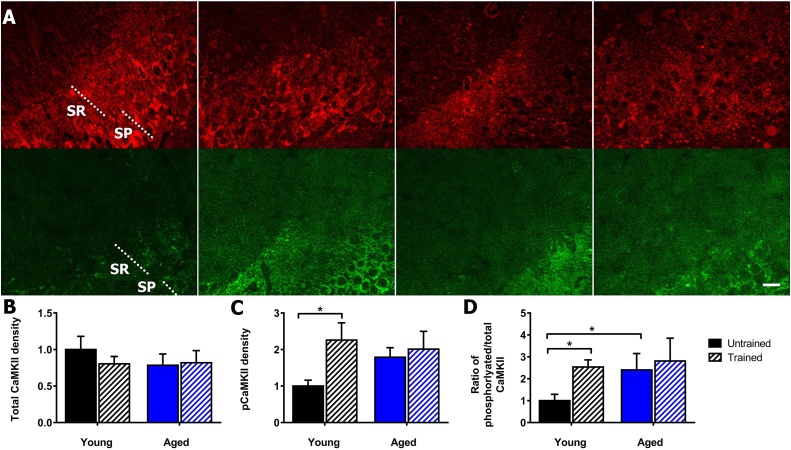
Age-dependent impairment of αCaMKII activation in stratum radiatum of hippocampal area CA3 after contextual fear conditioning. A) Representative images of phosphorylated and total αCaMKII in the stratum radiatum (SR) and stratum pyramidal (SP) of the CA3 hippocampus. Total αCaMKII stained using dsRed (red), phosphorylated αCaMKII stained using eGFP (green). Young untrained (far left), young trained (centre left), aged untrained (centre right), aged trained (far right), scale 20 μm. B) Contextual fear conditioning does not alter total αCaMKII levels in the stratum radiatum of the CA3 hippocampus in young and aged mice. C) Contextual fear conditioning induced an increase in autophosphorylation of αCaMKII in young mice (p = 0.025), but there was no significant change in aged mice. D) Contextual fear conditioning induced an increase in the ratio of autophosphorylated to total αCaMKII in young (p = 0.012), but not aged mice. Ageing induced an increase in ratio of phosphorylated:total αCaMKII in untrained mice (p = 0.033). Mean ± standard error of mean, * (p < 0.05). (For interpretation of the references to colour in this figure legend, the reader is referred to the web version of this article.)

The total αCaMKII in the CA3 stratum radiatum of the hippocampus (Fig. 2A), showed no significant differences by two-way ANOVA for training (F_(1,15)_ = 0.28, *p* = 0.60), aging (F_(1,15)_ = 0.43, *p* = 0.52) and interaction of training and aging (F_(1,15)_ = 0.58, *p* = 0.46) (Fig. 2B). The phosphorylated αCaMKII (Fig. 2A), showed significant differences for training (F_(1,11)_ = 4.9, *p* = 0.05) but not for ageing (F_(1,11)_ = 0.7, *p* = 0.44) or interaction of training and aging (F_(1,11)_ = 2.4, *p* = 0.15) (Fig. 2C). For the ratio of phosphorylated:total αCaMKII, training was significant (F^[1,16]^ = 5.0, *p* = 0.04), but not for aging (F_(1,16)_ = 2.2, *p* = 0.16) or interaction (F_(1,16)_ = 3.3, *p* = 0.09) (Fig. 2D). Tukey's post hoc analysis showed increases to phosphorylated αCaMKII after contextual fear training in young mice (*p* = 0.03) but not aged mice (*p* = 0.64) (Fig. 2C). The ratio of phosphorylated:total αCaMKII after training significantly increased in young mice (*p* = 0.01) but not in aged mice (*p* = 0.77). However, this ratio was significantly increased in aged when compared to young untrained mice (*p* = 0.03) (Fig. 2D) (Table 2).

**Table 2:** Mean and S.E.M. of total, phosphorylated and phosphorylated:total αCaMKII values for young untrained (YU), young trained (YT), aged untrained (AU) and aged trained (AT) mice groups in the CA3 hippocampus, lateral (LA), central (CE) and basolateral (BLA) amygdala.

	Mean Total (S.E.M.)								Mean Phosphorylated (S.E.M.)								Mean Phosphorylated:Total (S.E.M.)							
	YU		YT		AU		AT		YU		YT		AU		AT		YU		YT		AU		AT	
CA3	1.0	(0.2)	0.8	(0.1)	0.8	(0.2)	0.8	(0.2)	1.0	(0.2)	2.2	(0.5)	1.8	(0.3)	2.0	(0.5)	1.0	(0.3)	2.5	(0.3)	2.4	(0.7)	2.8	(1.0)
LA	1.0	(0.1)	0.5	(0.0)	0.7	(0.1)	0.5	(0.1)	1.0	(0.2)	1.3	(0.2)	1.4	(0.6)	1.3	(0.3)	1.0	(0.3)	2.3	(0.3)	2.4	(1.1)	2.5	(0.8)
CE	1.0	(0.2)	0.7	(0.1)	0.8	(0.1)	0.7	(0.1)	1.0	(0.2)	1.1	(0.5)	1.5	(0.5)	1.1	(0.1)	1.0	(0.3)	1.6	(0.5)	1.6	(0.5)	1.3	(0.2)
BLA	1.0	(0.1)	0.8	(0.1)	0.9	(0.1)	0.7	(0.0)	1.0	(0.2)	1.0	(0.1)	1.0	(0.1)	1.1	(0.1)	1.0	(0.3)	1.1	(0.2)	1.1	(0.2)	1.3	(0.1)

**Fig. 3:**
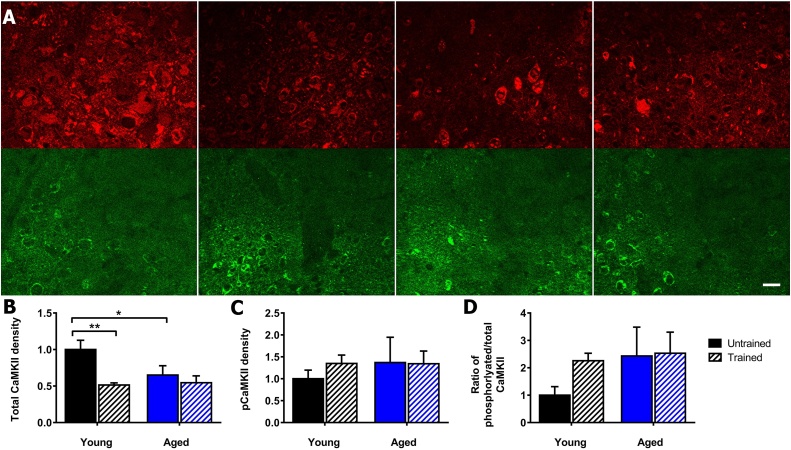
αCaMKII autophosphorylation in lateral amygdala in young and aged mice after contextual fear conditioning. A) Representative images of phosphorylated and total αCaMKII in the lateral amygdala. Total αCaMKII stained using dsRed (red), phosphorylated αCaMKII stained using eGFP (green). Young untrained (far left), young trained (centre left), aged untrained (centre right), aged trained (far right), scale 20 μm. B) Contextual fear conditioning significantly decreased total αCaMKII levels in the lateral amygdala in young (p = 0.004) but not aged mice. Ageing reduced the levels of total αCaMKII in untrained mice (p = 0.029). C) Contextual fear conditioning does not alter αCaMKII autophosphorylation in young and aged mice. D) Contextual fear conditioning does not alter the ratio of phosphorylated:total αCaMKII in the lateral amygdala in young and aged mice, although there was a non-significant trend of a conditioning-induced up-regulation in young age. Mean ± standard error of mean. (For interpretation of the references to colour in this figure legend, the reader is referred to the web version of this article.)

The total αCaMKII in the lateral amygdala (Fig. 3A), showed significant differences by two-way ANOVA for training (F_(1,16)_ = 8.3, *p* = 0.01), but not for aging (F_(1,16)_ = 2.4, *p* = 0.14) or interaction of training and aging (F_(1,16)_ = 3.4, *p* = 0.08) (Fig. 3B). The phosphorylated αCaMKII (Fig. 3A), showed no significant differences for training (F_(1,13)_ = 0.2, *p* = 0.64), ageing (F_(1,13)_ = 0.3, *p* = 0.60), and interaction of training and aging (F_(1,13)_ = 0.3, *p* = 0.59) (Fig. 3C). For the ratio of phosphorylated:total αCaMKII, there was also no significant effect of training (F_(1,16)_ = 3.9, *p* = 0.07), ageing (F_(1,16)_ = 1.1, *p* = 0.30) and interaction (F_(1,16)_ = 1.2, *p* = 0.28) (Fig. 3D) (Table 2). The non-significant trend for an up-regulation of phosphorylated:total αCaMKII after conditioning in young mice is consistent with the detection of an up-regulation, using immuno-electronmicroscopy (Rodrigues et al., 2004).

**Fig. 4:**
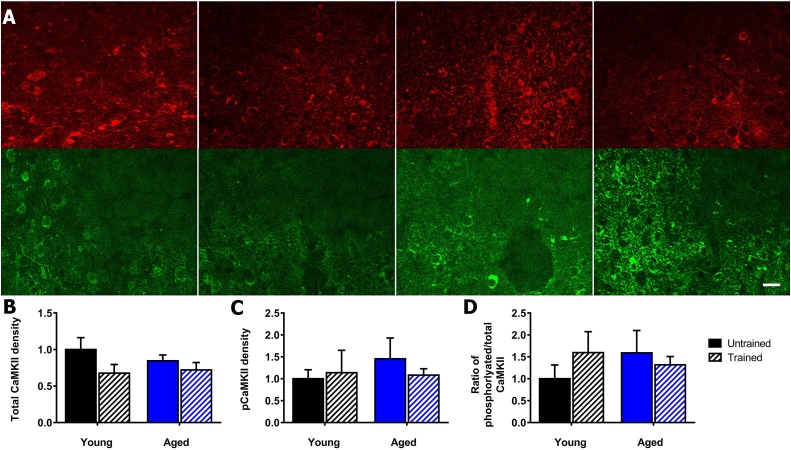
αCaMKII autophosphorylation in central amygdala in young and aged mice after contextual fear conditioning. A) Representative images of phosphorylated and total αCaMKII in the central amygdala. Total αCaMKII stained using dsRed (red), phosphorylated αCaMKII stained using eGFP (green). Young untrained (far left), young trained (centre left), aged untrained (centre right), aged trained (far right), scale 20 μm. B) Contextual fear conditioning does not alter total αCaMKII levels in the central amygdala in young and aged mice. C) Contextual fear conditioning does not alter αCaMKII autophosphorylation in young and aged mice. D) Contextual fear conditioning does not alter the ratio of phosphorylated:total αCaMKII in the central amygdala in young and aged mice. Mean ± standard error of mean. (For interpretation of the references to colour in this figure legend, the reader is referred to the web version of this article.)

The total αCaMKII in the central amygdala (Fig. 4A), showed no significant differences by two-way ANOVA for training (F_(1,16)_ = 3.5, *p* = 0.08), aging (F_(1,16)_ = 0.2, *p* = 0.65) and interaction of training and aging (F_(1,16)_ = 0.7, *p* = 0.42) (Fig. 4B). The phosphorylated αCaMKII (Fig. 4A), showed no significant differences for training (F_(1,11)_ = 0.1, *p* = 0.72), aging (F_(1,11)_ = 0.4, *p* = 0.54) and interaction of training and aging (F_(1,11)_ = 0.6, *p* = 0.44) (Fig. 4C) (Table 2). For the ratio of phosphorylated:total αCaMKII, there was also no significant effect of training (F_(1,16)_ = 0.6, *p* = 0.44), ageing (F_(1,16)_ = 0.7, *p* = 0.41) and interaction (F_(1,16)_ = 1.2, *p* = 0.29) (Fig. 4D) (Table 2).

**Fig. 5:**
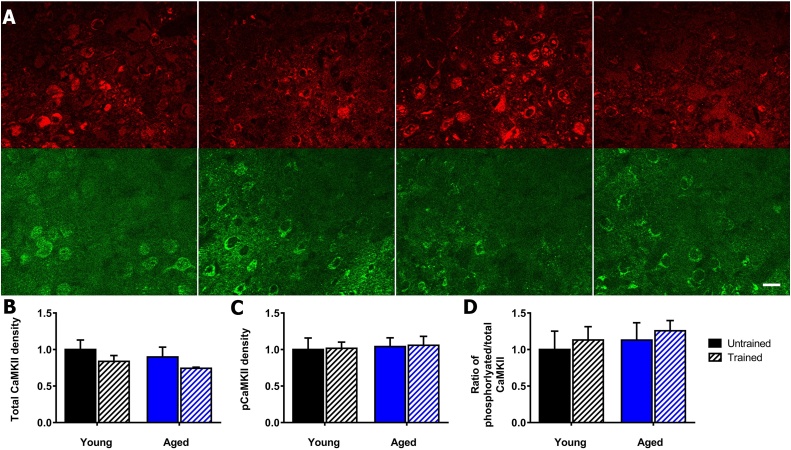
αCaMKII autophosphorylation in basolateral amygdala in young and aged mice after contextual fear conditioning.A) Representative images of phosphorylated and total αCaMKII in the basolateral amygdala. Total αCaMKII stained using dsRed (red), phosphorylated αCaMKII stained using eGFP (green). Young untrained (far left), young trained (centre left), aged untrained (centre right), aged trained (far right), scale 20 μm. B) Contextual fear conditioning does not alter total αCaMKII levels in the basolateral amygdala in young and aged mice. C) Contextual fear conditioning does not alter αCaMKII autophosphorylation in the basolateral amygdala in young and aged mice. D) Contextual fear conditioning does not alter the ratio of phosphorylated:total αCaMKII in the basolateral amygdala in young and aged mice. Mean ± standard error of mean. (For interpretation of the references to colour in this figure legend, the reader is referred to the web version of this article.)

The total αCaMKII in the basolateral amygdala (Fig. 5A), showed no significant differences by two-way ANOVA for training (F_(1,16)_ = 2.4, *p* = 0.14), aging (F_(1,16)_ = 0.9, *p* = 0.35) and interaction of training and aging (F_(1,16)_ < 0.1, *p* = 0.97) (Fig. 5B). The phosphorylated αCaMKII (Fig. 5A), showed no significant differences for training (F_(1,13)_ = 0.02, *p* = 0.90), ageing (F_(1,13)_ = 0.09, *p* = 0.76) and interaction of training and aging (F_(1,13)_ < 0.001, *p* = 0.99) (Fig. 5C). For the ratio of phosphorylated:total αCaMKII, there was also no significant effect of training (F_(1,13)_ = 0.39, *p* = 0.54), ageing (F_(1,16)_ = 0.4, *p* = 0.55) and interaction (F_(1,13)_ = 0.01, *p* = 0.99) (Fig. 5D) (Table 2).

## 4 Discussion

Experience-dependent molecular changes underlying synaptic plasticity in learning and memory can be modified by aging. An accurate and detailed understanding of how these molecules play a crucial role in hippocampus-dependent memory tasks and how they are affected by aging are poorly understood. We investigated this issue by looking at levels of a critical molecular step in induction of LTP, αCaMKII autophosphorylation at T286, which persists for some time after LTP induction (Irvine et al., 2006), in the hippocampus and amygdala in young and aged mice after contextual fear conditioning. For these experiments we compared the levels of autophosphorylation of αCaMKII and the ratio of phosphorylated:total αCaMKII. We used the ratio of phosphorylated:total αCaMKII to demonstrate the proportion of available αCaMKII that was autophosphorylated. As this would serve as a good marker in determining any increases in protein activity at any one point in time, considering any changes to the level of total αCaMKII after training or aging. It might also be a proxy for calcium signalling (Pasek et al., 2015).

Our experiments showed that contextual fear conditioning increases the levels of both phosphorylated and the ratio of phosphorylated:total αCaMKII in the hippocampal CA3 stratum radiatum in young, but not aged mice. To the best of our knowledge, this finding represents the first experiment conducted to determine the changes to αCaMKII autophosphorylation in the CA3 region after CFC. Previous studies have shown by blocking autophosphorylation of αCaMKII, contextual fear and spatial memories are impaired in mice (Irvine et al., 2005, Need and Giese, 2003). Our study suggests that an increase in phosphorylated αCaMKII after training in the CA3 region of the hippocampus may contribute to contextual fear memory formation in young age. Thus, in young mice the memory mechanism in the CA3 region after training might be associated with NMDA receptor-dependent LTP as suggested in a previous trace eyeblink conditioning study (Gruart et al., 2015).

Surprisingly, we found that in young, but not aged mice, total αCaMKII levels decreased in the lateral amygdala after CFC. The mechanism underlying this decreased expression is unclear. Interestingly, the decrease in αCaMKII levels is associated with a trend of an increase in phosphorylated:total αCaMKII in young mice, which is consistent with the detection of an up-regulation, using immuno-electronmicroscopy (Rodrigues et al., 2004). Our results indicate that this up-regulation does not occur in old age.

Aged mice which underwent CFC training, showed no differences for both levels of phosphorylated and total αCaMKII in the hippocampus and amygdala. This lack of change could be attributed to age-related difficulties in the induction and maintenance of LTP through an increased threshold for induction and difficulties in maintaining synaptic plasticity (Murphy et al., 2004, Bonhaus et al., 1990, Kumar, 2015, Deupree et al., 1993, Moore et al., 1993, Diana et al., 1995, Barnes et al., 1996, Kumar et al., 2007). Therefore, in line with our expectations there were no difference in phosphorylated αCaMKII and the ratio of autophosphorylation to total levels of αCaMKII after training in aged mice for all brain regions examined. This finding also suggests that it would be unlikely for VGCC-dependent LTP to occur after contextual fear conditioning in aged mice, as this would otherwise lead to an increase in the level of phosphorylated αCaMKII.

Interestingly, we found that for untrained mice, aging increased the ratio of phosphorylated:total αCaMKII in the CA3 stratum radiatum. This increase in baseline autophosphorylation may be due to the well-known increase in calcium entry through VGCC (Oh et al., 2016). The effect of such raised calcium levels in aged mice could be even further amplified by the depolarisation of post-synaptic spines which would lead to NMDA receptor activation and increased calcium influx, causing more autophosphorylation of αCaMKII. This persistent calcium elevation, could be a reason as to why training does not increase phosphorylated αCaMKII any further in aged mice, where a ceiling effect may have been reached. This is when the maximal level of phosphorylation at any point in time is reached and therefore any extra training-induced stimuli will not see any further increase to autophosphorylation.

In summary, our findings suggest that the hippocampus makes a unique contribution to contextual fear memory formation. Our results lead us to suggest that in young, but not old age NMDA receptor-dependent LTP in hippocampal area CA3 may contribute to contextual fear memory formation.

### Declaration of interest

Authors declare no conflict of interest.
